# Clinico-Radiological Presentation and Management of Carotid-Cavernous Fistulae: Real-Time Institutional Experience From Pakistan

**DOI:** 10.7759/cureus.106947

**Published:** 2026-04-13

**Authors:** Muhammad Naveed Majeed, Faiqa I Khan, Syed Haider Hassan, Ahsan Sarwar, Muhammad Jamil, Waleed Q Bashir, Haseeb Mehmood Qadri, Mahroona Fatima Khalid, Sundas Irshad, Asif Bashir, Qasim Bashir

**Affiliations:** 1 Neuroendovascular Surgery and Neurological Surgery, Punjab Institute of Neurosciences, Lahore, PAK; 2 Neurological Surgery, Punjab Institute of Neurosciences, Lahore, PAK; 3 Medical School, Shalamar Medical and Dental College, Lahore, PAK; 4 Neuroendovascular Surgery, Punjab Institute of Neurosciences, Lahore, PAK; 5 General Surgery, Lahore General Hospital, Lahore, PAK; 6 Neuroendovascular Surgery and Neurology, Punjab Institute of Neurosciences, Lahore, PAK

**Keywords:** arteriovenous fistula, caroticocavernous fistula, carotid artery injuries, endovascular, neurosurgery, pakistan

## Abstract

Background: Carotid-cavernous fistula (CCF) is an abnormal communication between the carotid artery and the cavernous sinus. This communication can be either high-flow or low-flow. Depending on the distinct anatomy of the shunt, it has effects on the various neurovascular structures that lie within the cavernous sinus.

Objective: To evaluate the immediate outcomes of CCF patients undergoing surgical and endovascular management.

Methodology: This is a retrospective observational study conducted at the Departments of Neuroendovascular Surgery and Neurosurgery at Punjab Institute of Neurosciences, Lahore. Consecutive patients diagnosed with CCF, who underwent diagnostic evaluation or treatment at the institution between January 2023 and December 2025, were included. Descriptive statistics were applied to analyze characteristics and outcomes.

Results: Of the total 10 patients, the most common clinical presentation was proptosis (90%; n=9), followed by chemosis (40%; n=4), ophthalmoplegia (30%; n=3), and cranial nerve palsy (10%; n=1). The majority of these cases were high flow (80%; n=8), categorized as being Barrow A. Arterial feeders originated exclusively from the internal carotid artery (ICA) in 60% (n=6) of cases, while 10% (n=1) were from the external carotid artery (ECA). Anterior and posterior drainage of CCF was mediated by the superior ophthalmic veins (SOV) (40%; n=4) and inferior petrosal veins (IPV) (20%; n=2), respectively. The majority of patients in our case series underwent stent (PK Papyrus, Biotronik SE & Co. KG, Berlin, Germany) placement, i.e., 70% (n=7), followed by ICA ligation in 20% of cases (n=2). Postoperative imaging revealed a fistulous leak in 60% (n=6), complete obliteration in 10%, and mild flow in 1% (n=1). Clinical outcomes improved in 90% (n=9), and no post-procedural hemorrhage, infarction, or cranial nerve deficit was reported.

Conclusions: Most of the patients had neuro-ophthalmic manifestations. Direct-type CCFs were the common type in our small case series, affecting the male gender predominantly, and most cases were post-traumatic. The endovascular management of CCF using covered stent deployment (PK Papyrus) yields reasonable radiological obliteration of the fistula, improved visual outcomes, and minimal intraoperative and immediate postoperative complications.

## Introduction

Carotid-cavernous fistula (CCF) is a rare complication of head and face trauma and can also happen spontaneously. CCF is an abnormal communication formed between the internal carotid artery (ICA) cavernous segment (C4) and the cavernous venous sinus (CVS) [1]. The cavernous segment of the ICA is fixed, and sudden acceleration-deceleration force causes the wall to rupture. Arteries having high pressure as compared to veins cause the shunting of blood from the ICA to the CVS. This usually creates a high-flow direct fistula (Type A). Approximately up to 87.17% results from craniofacial trauma [2]. Direct fistulas account for 70-90% of all CCF [3].

CCF can present with headache, hemorrhage, pulsating exophthalmoses, chemosis, or ophthalmoplegia [4]. As the cavernous sinus drains the orbit, catastrophic visual acuity deterioration can occur due to increased intraocular pressure [5]. Computerized tomographic angiography (CTA) or magnetic resonance angiography (MRA) can provide useful information regarding vascular structures, but digital subtraction angiography (DSA) is the gold standard [5]. 

Urgent occlusion of the fistula from circulation is necessary to prevent permanent vision loss. Minimal invasive endovascular treatment is now preferred due to its high success rate (75-88%) [5]. Endovascular options include embolization, stenting, coiling, or ballooning [5,6]. Rarely, they might complicate into a thromboembolic event, worsening of ocular symptoms of a pseudoaneurysm. This study aimed to evaluate the outcomes of CCF patients undergoing surgical and endovascular management at the Punjab Institute of Neurosciences.

## Materials and methods

This retrospective observational study was conducted at the Punjab Institute of Neurosciences, Lahore, Pakistan, a major tertiary-care referral center providing advanced neuroendovascular and neurosurgical services. The study period extended from January 2023 through December 2025. A non-probability, consecutive sampling approach was applied to include all patients diagnosed with CCF during the study period.

Inclusion criteria

Angiographically confirmed diagnosis of CCF using DSA, who underwent diagnostic evaluation or treatment at our center, were included. 

Exclusion criteria

Cases with missing or incomplete medical or imaging records, patients with prior definitive treatment for CCF at an outside institution, and patients with the absence of angiographically verified fistulous communication were excluded.

Diagnostic workup

DSA served as the primary tool for diagnosis, classification, and assessment of vascular anatomy. Fistulas were categorized using the Barrow classification system (Types A-D). Type A was direct, high-flow fistulas directly between the ICA and the cavernous sinus. Types B, C, and D were all indirect, low-flow fistulas. In Type B, the dural ICA communicates with the cavernous sinus; in Type C, the external carotid artery (ECA); and in Type D, both ICA and ECA branches communicate with the cavernous sinus [7]. Supplementary imaging, including CTA, MRI/MRA, and orbital ultrasonography, was reviewed, when available, to evaluate venous congestion, arterial feeders, and orbital involvement.

Data collection procedure

Data were retrospectively extracted from medical charts, radiology databases, and procedural logs using a standardized template. Variables collected included demographics (age and gender), clinical characteristics (trauma history, comorbidities, symptom profile, duration, and laterality), imaging features (Barrow type, arterial supply, venous drainage pattern, cortical venous reflux, and associated pseudoaneurysm), management details (intervention type, device specifications, embolic materials, adjunctive techniques, and complications), and outcomes (angiographic obliteration status, symptom improvement, recurrence, new neurological deficits, and mortality).

Intervention protocol

Management strategies were personalized based on fistula type, symptom severity, arterial inflow patterns, venous drainage, and operator judgment. Endovascular therapies included covered stent deployment (e.g., PK Papyrus, Biotronik SE & Co. KG, Berlin, Germany), coil embolization, balloon-assisted techniques, and adjunctive liquid embolization when indicated. For endovascular cases, a 4 cc Aggrastat bolus was given, then loaded with Co-Plavix 300 mg after the procedure (if not loaded preoperatively). Surgical ICA ligation was performed selectively for patients with sufficient collateral circulation when endovascular options were limited.

Follow-up and outcome assessment

Clinical follow-up was performed after treatment and during subsequent outpatient visits. Improvement in proptosis, chemosis, ophthalmoplegia, visual acuity, and cranial nerve function was recorded. Follow-up imaging (DSA, CTA, or MRI) was obtained at the third month after intervention, as required to assess fistula obliteration.

Statistical analysis

Data was entered into a Microsoft Excel (Microsoft Corporation, Redmond, Washington, United States) spreadsheet and analyzed using IBM SPSS Statistics for Windows, Version 27 (Released 2019; IBM Corp., Armonk, New York, United States). Descriptive statistics were applied to summarize patient demographics and clinical characteristics. Continuous variables were presented as mean and standard deviation if normally distributed or median and interquartile range if not. Categorical variables were reported as frequencies and percentages. All data were anonymized before analysis to ensure patient confidentiality. Only authorized members of the research team had access to the full dataset. 

Ethical approval was obtained from the Institutional Review Board of the Punjab Institute of Neurosciences via reference #2221, dated June 19, 2025. Patient confidentiality was ensured by anonymizing identifiers during data handling and manuscript preparation.

## Results

Of the total ten patients in our case series, there were six males (60%) and four females (40%), with a mean age of 37.2±16.5 years. Of these, three (30%) were hypertensive, three were smokers (30%), and one was diabetic (10%). A history of trauma was noted in six (60%); however, no patient gave a history of any previous surgery (Table 1).

**Table 1:** Patients' demographics, clinicopathological features, management, and clinical and radiological outcomes. ICA: internal carotid artery, ECA: external carotid artery, CCF: carotid-cavernous fistula PK Papyrus (Biotronik SE & Co. KG, Berlin, Germany).

Sr.	A/G	Barrows Classification	Symptoms	Management	Imaging Outcomes	Visual Outcomes
1	40/F	Left, Type A	Proptosis, chemosis	Left cavernous covered stent PK Papyrus 4mmx15mm	Faint fistulous blush	Improved
2	18/M	Right, Type A	Proptosis, ophthalmoplegia	Right cavernous covered stent PK Papyrus 4.5mmx15mm	Faint fistulous blush	Improved
3	40/M	Left, Type A	Proptosis	Balloon-mounted covered coronary stents were placed in the left ICA cavernous segment. (PK Papyrus 4.00mm x 20mm distally and 4.0mm x 15mm proximally)	Mild flow reduction across the fistula	Improved
4	15/M	Right, Type A	Chemosis, ophthalmoplegia	Balloon-mounted covered coronary stents were placed in the left ICA cavernous segment, along with partial coiling of the pseudoaneurysm	Complete obliteration	Unchanged status
5	30/F	Left, type A	Proptosis	Left ICA cavernous segment covered stent PK Papyrus 3.5mmx20mm	Complete Obliteration	Improved
6	46/M	Right, Type A	Cranial nerve palsies, proptosis	Right ICA Ligation	Faint fistulous blush	Improved
7	35/F	Right, Type A	Diplopia, proptosis	Right ICA ligation	Faint fistulous blush	Improved
8	75/F	Right, Type A	Proptosis, chemosis, ophthalmoplegia	Right cavernous covered stent PK Papyrus 4.5mmx15mm and Right ICA ligation	Faint fistulous blush	Improved
9	40/M	Left, Type B	Proptosis	Left ICA cavernous segment covered stent PK Papyrus 3.5mmx20mm	Faint fistulous blush	Improved
10	30/M	Left, Type C	Proptosis, chemosis	Direct coiling of left indirect CCF Type C with complete obliteration and coil implant volume 50cm^3^	Complete obliteration	Improved

**Figure 1:**
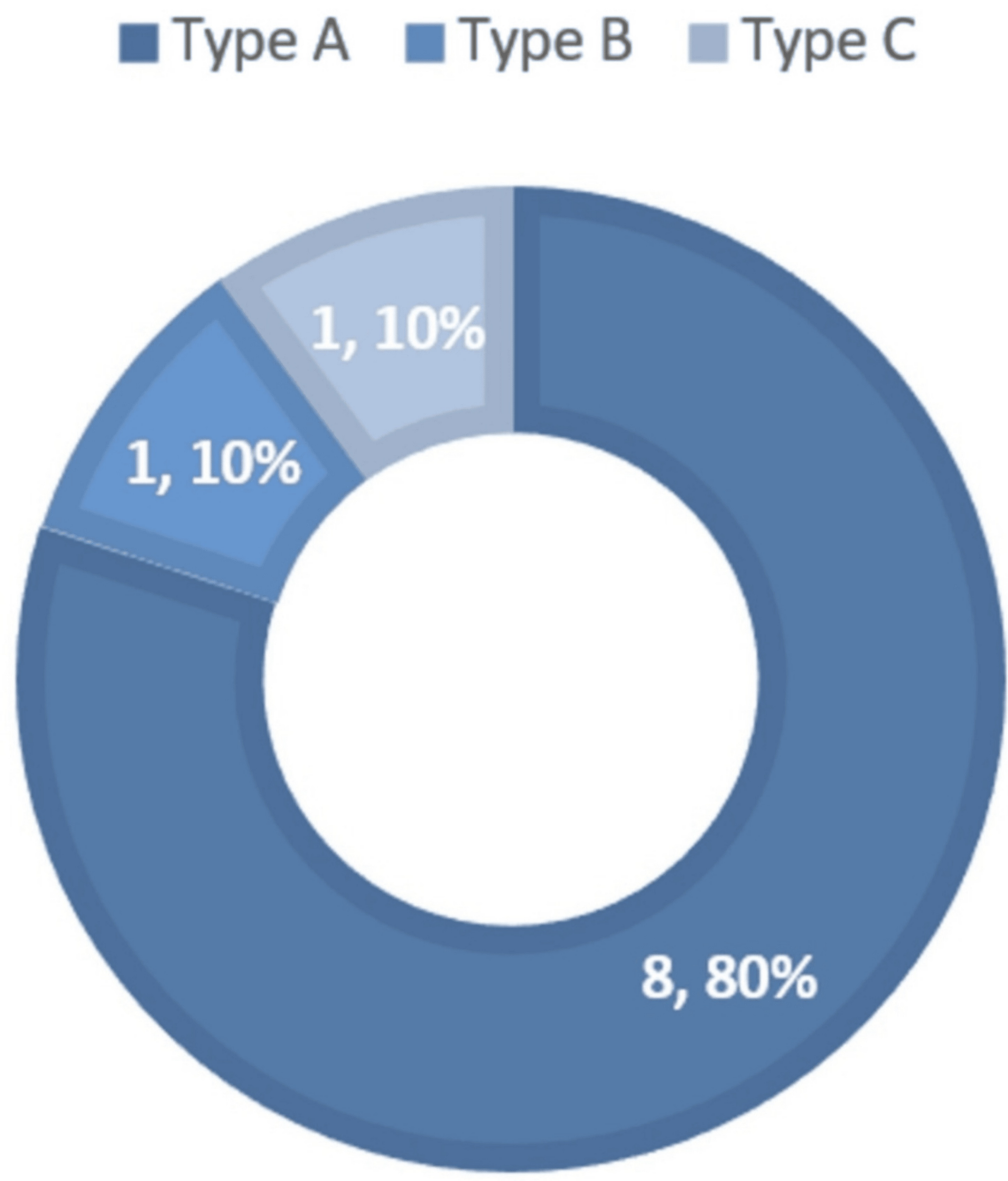
Barrow classification of carotid-cavernous fistulae in our case series, with frequency followed by percentage occurrence (%), where N = 10.

The mean duration of onset of symptoms was 8±4.5 months. The most common clinical presentation was proptosis, occurring in nine (90%), followed by chemosis in four (40%), ophthalmoplegia in three (30%), and cranial nerve palsy in one (10%). The CCF classification according to the Barrow classification is provided in Figure 1.

**Figure 2:**
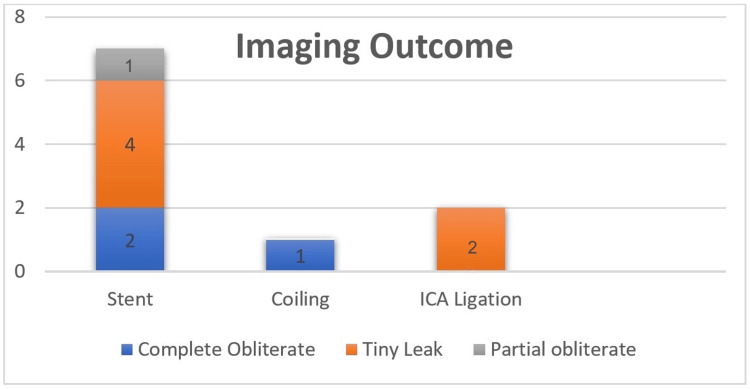
Type of management and imaging outcome, where N = 10. ICA: internal carotid artery

Most of the CCF in our case series were high flow, i.e., direct in eight (80%); one (10%) was Type A; and the rest (10%) were Type B. There was no variation in laterality of disease; five (50%) were left-sided, and five (50%) were right-sided lesions. Arterial feeders originated exclusively from the ICA or its branches in six (60%), from the ECA in one (10%), from posterior circulation in one (10%) via the posterior cerebral artery, and mixed feeding from branches of both the ICA and ECA in one (10%) and from anterior and posterior circulation in one (10%). Anterior drainage of CCF in our case series was mediated by the superior ophthalmic vein (SOV) exclusively in four (40%), posterior drainage by the inferior petrosal vein (IPV) exclusively in 20%, and mixed drainage patterns (both SOV and IPV) were observed in four (40%). Cortical reflux was seen in seven (70%). About seven (70%) of patients in our case series underwent stent placement (Figure 2).

**Figure 3:**
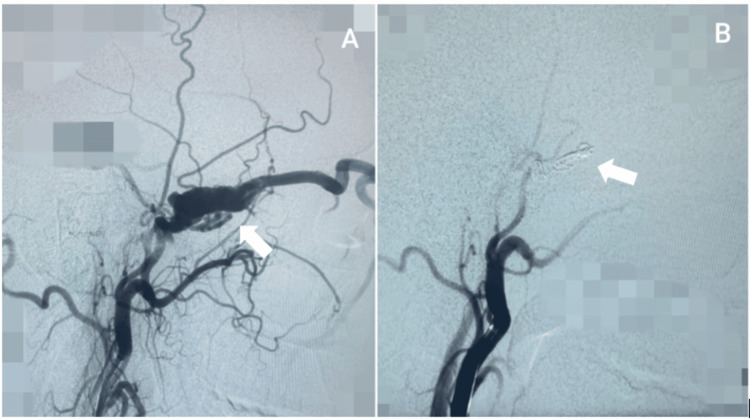
A 30-year-old male presented with left-sided indirect carotid-cavernous fistula (CCF). A. pre-operative digital subtraction angiography shows indirect fistula Type B - arrow indicating external carotid artery meningeal fistula with cavernous sinus; B: post-operative digital subtraction angiography shows external carotid artery branch embolization with coils (arrow) reinforced with glue. No post-embolization fistula noted.

**Figure 4:**
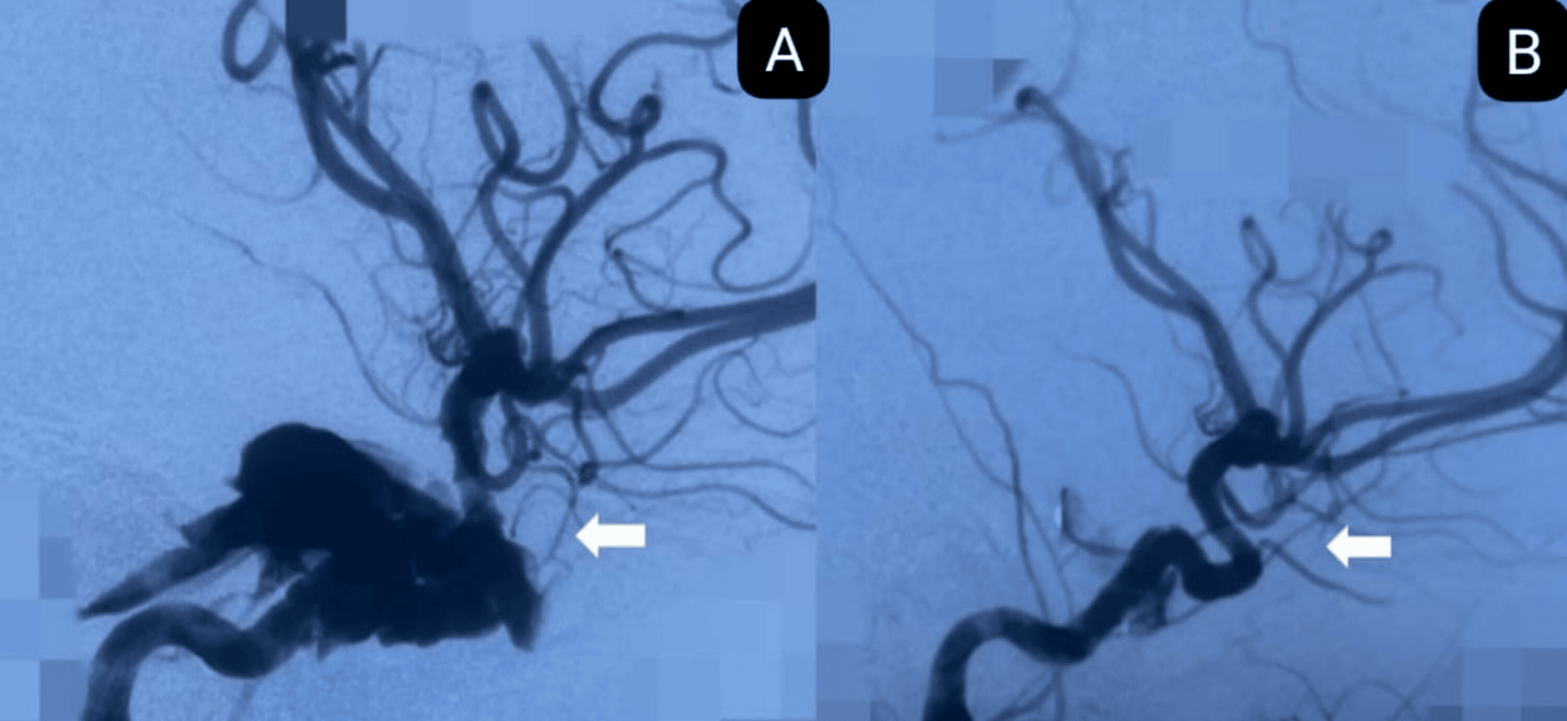
A 30-year-old female presented with a left direct carotid-cavernous fistula (CCF) Type A. A: pre-operative digital subtraction angiography showing fistula between the cavernous segment of the left internal carotid artery and the left cavernous sinus (arrow)- direct Type A fistula; B: post-operative digital subtraction angiography, arrow indicating complete obliteration of fistula after cover stent placement.

Post-operative imaging revealed a tiny fistula leak in six; immediate complete obliteration was achieved in three, and partial obliteration in one patient. Clinical outcomes improved in 90% of patients, with no post-procedural complications (hemorrhage, infarction, cranial nerve deficit), recurrence, or mortality. Two illustrative cases are shown in Figures 3, 4.

## Discussion

The reported yearly incidence of CCF is 0.37 per 100,000 people [1]. Unfortunately, incidence in our setting is not well-reported. Rahmatian et al., in their meta-analysis, reported 51.95% indirect and 48.05% direct CCF cases; additionally, a history of trauma was documented in 87.17% of cases [2]. In our series, the majority (80%) of cases had high-flow direct (Barrow’s Type A) fistulae. CCF is a reported complication of craniofacial trauma (0.17-0.27%), with the incidence being 4% of all cerebrovascular injuries [4]. Most commonly, with a base of skull fracture, the middle fossa is 8.3%, the anterior fossa is 2.4%, and the posterior fossa is 1.7% [4]. In our series, six (60%) had a history of trauma, and we could not find the concurrent skull base pathology. The mean age in our series was 37.2±16.5 years. The current scientific literature is also suggestive of trauma being the most common (87.17%) cause, followed by the spontaneous fistulous formation (10.3%), with a mean age of 48.10 years [2]. There is no significant gender predilection [2]. If trauma is considered the most reported etiology, then young-adult males are more prone to developing CCF.

The most common presentation in our series was proptosis in nine (90%). CCF results in the disruption of venous drainage and resultant retrograde flow in ophthalmic veins [5]. This engorgement leads to fluid leakage into the orbital interstitium, resulting in accumulation of fluid in connective tissue and hence leads to proptosis. This also increases the intraocular pressure (IOP). IOP can cause optic nerve compression, which could lead to permanent optic atrophy. Malik et al. (case series from Pakistan) and Al-Shalchy et al. (systematic review from Iraq) report proptosis as the most common presenting symptom in 18 (100%) and 58 (14.39%), respectively [5,6]. A detailed neuro-ophthalmological examination (including examination for exophthalmoses, chemosis, cranial nerve (2, 3, 4, 6) examination, IOP, visual acuity, and fundoscopy) is necessary to rule out CCF. In a meta-analysis by Rahmatian et al., chemosis was the most common presenting symptom, 84% [2]. Early chemosis (edema of the conjunctiva) can be easily mistaken for conjunctivitis (which is the inflammation of the conjunctiva). Other manifestations include bruit (75%), orbital pain (31%), and ophthalmoplegia (11%), findings that are somewhat similar to our case series [2]. Rare symptoms may include tinnitus, epistaxis, and vertigo [1]. Ma et al. reported a case of CCF who presented with quadriplegia [8]. In his case, drainage was via the superior petrosal sinus vein and the lateral mesencephalic vein into the cervical spinal cord veins. Venous drainage-based classifications explain the patient’s symptomatology and guide the management plan. Thomas et al. proposed a classification of CCF into five categories: posterior/inferior drainage; posterior/inferior and anterior drainage; anterior drainage only; drainage into cortical veins; and high flow shunts (Barrows Type A) ± multiple routes [9]. Wherein, posterior includes the superior petrosal vein (SPV) and IPV, the parapharyngeal, and the pterygoid plexus. Anterior includes SOV and IOV. Cortical veins include superficial cerebral, cerebellar, and perimesencephalic veins. In our series, cortical reflux was seen in 70%.

Considering trauma as the leading cause of CCF, bilateral presentations are rare, 1-2%, and there is no significant conclusion on laterality [10]. Bilateral presentations are often seen in indirect cases where a predisposing factor is any systemic illness. However, in our series, we did not find any bilateral CCF cases. Spontaneous CCF can occur due to vessel weakness. Predisposing factors might include hypertension, atherosclerosis, diabetes, trauma during transsphenoidal surgery, Ehlers-Danlos, and fibromuscular dysplasia [10]. In our series, the prominent comorbidities were hypertension and smoking, three (30%).

Timely endovascular management is the key to recovery. Especially in direct fistulas, where endovascular management resulted in full recovery (82%) of visual symptoms [2]. Employed endovascular options are embolization via glue or coiling, stent, balloon, or flow diverter. The choice of agent to be used depends on vascular anatomy and the availability of expertise and resources. The procedure aimed to obliterate the fistulous route, maintaining the patency of the named vessels. 

In this series, PK Papyrus stents were deployed in seven (70%) patients; immediate complete obliteration was observed in two (28.60%), and a faint fistulous blush was observed in four cases (57.14%). However, symptoms were improved in six (85.70%). The PK Papyrus coronary stent is a single-layered, biocompatible, porous (polyurethane)-covered stent, which has superior durability and lower chances of thrombus formation as compared to older ones [11]. Seraj et al. in 2024 reported 75% immediate occlusion after BeGraft (Bentley InnoMed GmbH, Hechingen, Germany) covered stent deployment, with improvement of ocular symptoms in 4/4 patients having direct CCF [12]. Stents are superior, as they keep the vessel patent, simultaneously occluding the fistulous track. The exact size of the stent and correct deployment are necessary to ensure optimal results. Abramyan et al. also reported successful deployment of flow diverter stents (FDs), with 100% immediate obliteration on imaging [13]. It is theorized that stenting devices can promote vessel endothelium proliferation and, hence, aim to repair the defect with time [13]. Stents or coils can, however, complicate thrombus formation, which we did not encounter in any [14].

Surgical ligation of the ICA is also another option, where applicable. Cases where collateral circulation is sufficient (as per balloon test occlusion; BTO) and ligation will not harm the patient. We used ICA ligation in two cases. A tiny leak was observed in post-operative DSA in both; however, vision in both patients was improved postoperatively.

Another available option is stereotactic radiosurgery (SRS). SRS is suitable for recurrent cases or indirect fistulas, and its efficacy is even higher when prior embolization is done [15]. Indirect fistulas are more prone to resolve spontaneously [11,15]. However, embolization (63.2%) was preferred over coiling (57.8%) for dural (indirect) CCF [16,17]. We used coiling for indirect CCF successfully, with complete obliteration and vision improvement in all cases. Nurimanov et al. showed the resolution of symptoms in 80.9% and partial resolution in 16.7% of patients in which coils were used [18]. Coil embolization is currently considered the first-line treatment [13]. Moreover, Texakalidis et al. in a meta-analysis showed the complete obliteration of 95.68% of CCF with balloon embolization and 92.59% with coil embolization [15]. We did not use ballooning alone in any patient. Detachable balloons are more prone to premature deflation or rupture. Meanwhile, coils are easy to control and install [14]. However, in a Pakistani case series by Malik et al. in 2024, balloon embolization was successful in 83.3% with incomplete occlusion in two patients [6]. This highlights the expertise developed from in-hand tools in a resource-limited setting. Coils are also used in conjunction with glue (EVAL-I, Merit Medical Systems, Utah, USA, or Onyx, Medtronic, Dublin, Ireland) in selected cases to achieve complete obliteration, as demonstrated by Yang et al. in 2025 [19]. In our study, we used a balloon-assisted stent along with coiling (for pseudoaneurysm). In one patient, a tiny leak was noted, but symptoms improved.

Limitations

Data was collected from a single institute; even though our institute gets referrals from all over the country, it still does not represent the entire geographical area (Pakistan). Due to limited sample size, larger prospective studies should be done to assess the role of endovascular management, especially cost-effective stents vs the surgical management. In addition, it increases the likelihood of a larger proportion of patients having underlying background issues, leading to CCF. Data was collected retrospectively, so it can create a recall bias. Importantly, outcomes for CCF patients (following intervention) can vary, leading to difficulty in accurately assessing the extent of their recovery. For this reason, a standardized outcome scoring system is necessary. Such a system can help with better monitoring of symptoms and can also help with patient referrals and research collaborations between institutions. Post-operative patients often face short-term relief from symptoms; in the long run, many face recurrences or the appearance of new symptoms. Long-term surveillance helps monitor patients for signs of recurrence and also helps to assess the viability and effect of the administered treatment.

Recommendations

Early diagnosis in CCF cases is of utmost importance to prevent severe symptoms from manifesting, such as vision loss and cranial nerve deficits. Prompt intervention is recommended to prevent permanent damage. Clinicians should have a high index of suspicion when encountering these symptoms. Early referral to advanced centers will prevent deleterious complications. Moreover, for accurately assessing the extent of recovery, a standardized outcome scoring system is necessary. Such a system can help with better monitoring of symptoms and can also help with patient referrals and research collaborations between institutions. Where angiography is not available, the use of initial MRI is recommended, as it can allow early and non-invasive diagnosis. Use of advanced modalities like 3D rotational angiography for delineation of exact vascular anatomy and careful patient-based selection of tools.

## Conclusions

Direct-type CCFs were the common type in our small case series, affecting the male gender predominantly, and most cases were post-traumatic. In a resource-constrained setting like ours, the endovascular management of CCF using covered stent deployment (PK Papyrus) yields reasonable radiological obliteration of the fistula, improved visual outcomes, and minimal intra-operative and immediate post-operative complications.
